# Supplementary material for: The impact of demographic factors and personality traits on nurse compassion fatigue: A cross-sectional analysis
Source: PLoS One. 2025 Jul 31;20(7):e0329270. doi: 10.1371/journal.pone.0329270 (PMC12312956; doi:10.1371/journal.pone.0329270)
Supplement: Attachment — Submitted filename: Response to Reviewers.doc [file pone.0329270.s002.doc]

**Rebuttal Letter**

Manuscript Number: PONE-D-25-23186

Title: The impact of demographic factors and personality traits on nurse compassion fatigue: A cross-sectional analysis

Corresponding Author: Min Xie

Date: June 20

**Part 1: Response to Academic Editor**

Dear Dr. Alamri

We sincerely appreciate your editorial guidance and the opportunity to revise our manuscript. We have thoroughly addressed all points raised during the review process:

1. Manuscript Formatting & Language Polishing

The manuscript has been strictly reformatted according to the PLOS ONE style templates. Additionally, comprehensive linguistic revisions were performed to: (1)correct grammatical, typographical and ambiguity errors ;(2)ensure clarity and compliance with PLOS ONE's language standards ;(3)Validate terminology consistency. All modifications related to formatting and language refinement are highlighted in yellow in the 'Revised Manuscript with Track Changes'.

2.ORCID Validation

The corresponding author's ORCID (0000-0002-1876-7993) has been validated in Editorial Manager.

3.Code Sharing

No author-generated code was used in this study

4.Data Availability

The minimal dataset required to replicate our findings has been uploaded as Supporting Information files in the online submission system.

5.Author Affiliation

All authors are now linked to their respective affiliations in the manuscript.

6.Funding Statement

The authors received no specific funding for this work. This declaration has been entered in the online submission system and updated in the Funding Information section.

7.Image Consent

This study contains no figures depicting patients/participants, thus no consent forms are required.

8.Ethics Statement

The full ethics statement has been added to the Methods section (Highlighted in Yellow in Page 5-6, Line 131-140). Scanned copies of both the Ethical Review Opinion Form and its English translation have also been uploaded to the online submission system.

**Part 2: Response to Reviewers**

**Reviewer #1**

**Comment 1.** I suggest adding a subchapter entitled “Procedure” to the Methodology section, describing the research procedure from data collection to the present report.

**Response:** Thank you very much. We have revised this as suggested in the Methodology section (Highlighted in Yellow in Page 4-5, Line 104-114).

**Comment 2.** “Participant Characteristics” should be moved to the Methodology section.

**Response:** Thank you very much. While we appreciate the rationale underlying this recommendation, the disciplinary standards for reporting cross-sectional data—as demonstrated in the referenced PLOS ONE study (doi:10.1371/journal.pone.0324456)—support the inclusion of “Participant Characteristics”within Results sections. To meet PLOS ONE's style requirements, we maintain the original placement.

**Comment 3.** Please also add section and subsection numbering to make the paper easier to read for potential readers.

**Response:** Thank you very much.Aligning with PLOS ONE's style guidelines for hierarchical section labeling and the explicit formatting precedent established in the referenced PLOS ONE study（doi:10.1371/journal.pone.0323867）, we have systematically numbered all subsection headings throughout the Results section.(Highlighted in Yellow in Page 7-12, Line 197,213,236).

**Comment 4.** Furthermore, in terms of content, when discussing demographic factors, the specific characteristics of contemporary generations should be highlighted. Although this is not your leading variable, it falls within the broadly understood confounding variable. Therefore, I suggest adding a mention of contemporary generations in relation to demographic factors in the introduction. Consider referring to doi: 10.3389/fspor.2024.1416154

**Response:** Thank you very much.We acknowledge the conceptual merit of incorporating generational dimensions. However, cross-cultural validation gaps impede direct application of Western generational taxonomies to China's demographic landscape. Current scholarship lacks operationally valid generational cohorts specific to China's socioeconomic evolution. Consequently, we have deliberately reserved this aspect for critical examination in the Limitations section(Highlighted in Yellow in Page 17, Line 362-364).

**Reviewer #2**

**Comment 1.** The overall structure of the Methods section is clear; however, some areas need further improvement:It would strengthen the paper to briefly explain why a cross-sectional design and convenience sampling were chosen and to acknowledge their limitations.

**Response:** Thank you very much.We selected a cross-sectional design to efficiently capture how demographic factors and personality traits collectively influence compassion fatigue at a critical post-COVID-19-pandemic juncture, prioritizing timely evidence generation over longitudinal causality assessment. Convenience sampling maximized participation feasibility given nurses' unpredictable shift patterns amid hospital COVID-19 recovery initiatives. We have revised this as suggested in the Limitations section (Highlighted in Yellow in Page 17, Line 356-362).

**Comment 2.** The response rate of 92.6% is high, which is positive. However, it would be helpful to clarify how the sample size was determined for example, was a power calculation performed?

**Response:** Thank you very much.The sample size was calculated using G*Power version 3.1.9.7. We have revised this as suggested in the Methods section (Highlighted in Yellow in Page 5, Line 117-122).

**Comment 3.** Regarding the participants:Did they complete the survey during working hours or on their own time?Was participation completely voluntary and anonymous?

**Response:** Thank you very much.Questionnaire administration occurred during regular work shifts. Participants voluntarily completed the anonymous surveys on personal mobile devices immediately upon receipt, with no compensation provided for participation. We have revised this as suggested in the Methods section (Highlighted in Yellow in Page 7, Line 176-181).

**Comment 5.**The use of the Chinese versions of the CCF-SS and CNEO-FFI is appropriate.You’ve included reliability scores, which is good. However, the openness subscale shows a relatively low Cronbach’s alpha (0.60), so it may be worth briefly noting this as a limitation.

**Response:** Thank you very much. We have revised this as suggested in the Limitations section (Highlighted in Yellow in Page 17, Line 364-367).

**Comment 6.**The Discussion section presents relevant insights and draws helpful connections to prior research. However, I recommend revising this section for clarity, focus, and conciseness. While the content is valuable, the current version is too lengthy and at times repetitive, with detailed role descriptions that could be summarized. It would also be helpful to include a brief reflection on how compassion fatigue may impact patient care, and conclude with a clear summary of key findings and their practical implications.

**Response:** Thank you very much. We have revised the Discussion section to enhance conciseness while preserving its original structure and key insights. Specifically: （1）Streamlined each paragraph to eliminate redundancies.Reduced the word count from 1,412 to 662 （2）Added analysis on compassion fatigue's impact on patient care (Highlighted in Yellow in Page 16, Line 338-344) ；（3）Repositioned references 23 and 24 to ensure contextual coherence(Highlighted in Yellow in Page 19, Line 452-457) ；（4）Incorporated three new references (26-28) supporting clinical implications (Highlighted in Yellow in Page 19, Line 460-467).

We believe these revisions significantly strengthen the manuscript’s rigor and clinical relevance. Thank you for your time and consideration.

Sincerely,

Min Xie

The First Affiliated Hospital of Chengdu Medical College

redpinkblue@sina.com
